# The Prediction of LptA and LptC Protein–Protein Interactions and Virtual Screening for Potential Inhibitors

**DOI:** 10.3390/molecules29081827

**Published:** 2024-04-17

**Authors:** Yixin Ren, Wenting Dong, Yan Li, Weiting Cao, Zengshuo Xiao, Ying Zhou, Yun Teng, Xuefu You, Xinyi Yang, Huoqiang Huang, Hao Wang

**Affiliations:** 1 Key Laboratory of Mass Spectrometry Imaging and Metabolomics (Minzu University of China), National Ethnic Affairs Commission, Beijing 100081, China; moondryad@outlook.com; 2 Institute of National Security, Minzu University of China, Beijing 100081, China; 3 School of Pharmacy, Minzu University of China, Beijing 100081, China; 4 Beijing Key Laboratory of Antimicrobial Agents, Laboratory of Pharmacology, Institute of Medicinal Biotechnology, Chinese Academy of Medical Sciences and Peking Union Medical College, Beijing 100050, China; 5 Division for Medicinal Microorganism-Related Strains, CAMS Collection Center of Pathogenic Microorganisms, Beijing 100050, China; 6 State Key Laboratory of Bioactive Substances and Functions of Natural Medicines, Institute of Medicinal Biotechnology, Chinese Academy of Medical Sciences and Peking Union Medical College, Beijing 100050, China; 7 Key Laboratory of Ethnomedicine (Minzu University of China), Ministry of Education, Beijing 100081, China

**Keywords:** antibiotic resistance, gram-negative bacteria, lipopolysaccharide transport, CADD, protein–protein interaction

## Abstract

Antibiotic resistance in Gram-negative bacteria remains one of the most pressing challenges to global public health. Blocking the transportation of lipopolysaccharides (LPS), a crucial component of the outer membrane of Gram-negative bacteria, is considered a promising strategy for drug discovery. In the transportation process of LPS, two components of the LPS transport (Lpt) complex, LptA and LptC, are responsible for shuttling LPS across the periplasm to the outer membrane, highlighting their potential as targets for antibacterial drug development. In the current study, a protein–protein interaction (PPI) model of LptA and LptC was constructed, and a molecular screening strategy was employed to search a protein–protein interaction compound library. The screening results indicated that compound 18593 exhibits favorable binding free energy with LptA and LptC. In comparison with the molecular dynamics (MD) simulations on currently known inhibitors, compound 18593 shows more stable target binding ability at the same level. The current study suggests that compound 18593 may exhibit an inhibitory effect on the LPS transport process, making it a promising hit compound for further research.

## 1. Introduction

Infections resulting from antibiotic-resistant bacteria have emerged as one of the foremost threats to global public health and healthcare. In 2016, the British government published a comprehensive review report projecting that by 2050, antibiotic resistance will claim the lives of at least ten million people annually and impose a global economic burden of at least one quadrillion dollars [1]. In 2019, the Centers for Disease Control and Prevention (CDC) released the ‘Threats Report on Drug-Resistant Bacteria’, and in 2022, the Lancet published ‘Global Burden of Bacterial Antimicrobial Resistance in 2019: A Systematic Analysis’, both of which underscore the perils of antibiotic resistance [2,3].

Gram-negative (G-) bacteria, due to their outer membrane (OM) barriers, are generally considered difficult to treat and prone to developing resistances to multiple antibiotics. Furthermore, because such outer membrane barriers restrict the entry of drug molecules, drug discovery against G- bacteria is facing significant hurdles.

Currently, the latest drugs effective against Gram-negative bacteria include Cefiderocol, introduced in 2019, hailed as a “game-changer” in the fight against Gram-negative bacteria. However, fundamentally, it is still a third-generation cephalosporin antibiotic derived from the traditional antibiotic parent structure. Like the other third-generation cephalosporin antibiotics, Cefiderocol does not exhibit significant activity against some challenging G- bacteria like *Pseudomonas aeruginosa*. Furthermore, recent studies have reported that *Acinetobacter baumannii* has established the resistance to Cefiderocol [4,5].

**Figure 1:**
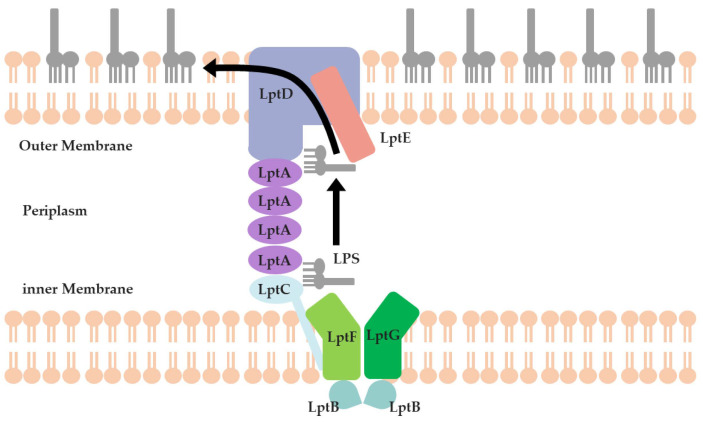
Schematic of the LPS transportation system.

Lipopolysaccharide (LPS) is an important component of the OM of G- bacteria. Therefore, disrupting the functionality of LPS can effectively hinder the formation of the OM and, ultimately, might help to achieve the goal of combating drug-resistant G- bacteria [6] (Figure 1). LPS undergoes assembly on the cytoplasmic membrane and is then transported to the OM for anchoring through the Lpt protein series (LptA to LptG). The complex of LptB, LptF, and LptG acts as an ATP-binding cassette (ABC) transporter to facilitate the extraction of LPS from the cytoplasmic membrane. LptC, binding directly to the LptBFG complex on the outer leaflet of the cytoplasmic membrane, serves as the docking partner for LptA [7]. LptA accepts the LPS from LptC and transports it across the periplasm through a twisted boat structure formed by β-folds. During this process, the Lpt proteins (LptC, LptA, and LptD) form a bridge spanning from the cytoplasmic membrane to the OM, then LPS can reach this bridge through LptC. By passing through several LptA proteins, LPS can ultimately enter the interior of LptD to complete the process of LPS translocation in the periplasm. Finally, the LptDE complex, located on the OM, translocates LPS across the OM and facilitates the assembly of LPS on the cell surface [8].

According to the LPS transport mechanism, LptA and LptC are considered key proteins responsible for transporting LPS within the periplasm [9,10]. Notably, they are conserved in G- bacteria without known homologous proteins in humans, which implies that designing drugs aimed at LptA and/or LptC could effectively minimize the non-specific effects on human hosts. It has been reported that inhibiting the interaction between LptA and LptC can effectively trigger antibacterial activity [11], which makes them important targets for antibacterial drug design. Furthermore, their effectiveness has been experimentally verified.

**Figure 2:**
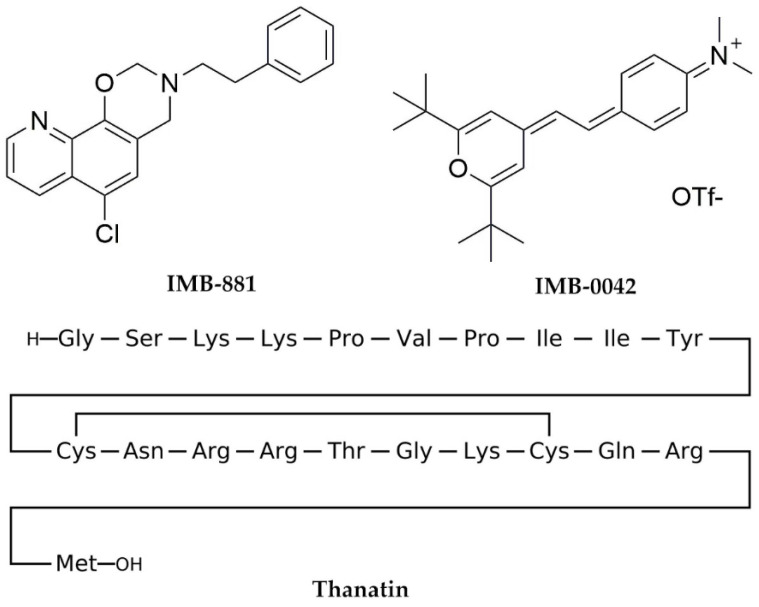
Inhibitors of the LptA–LptC interaction.

Currently, two molecular inhibitors (IMB-881 and IMB-0042) (Figure 2) that target the LptA and LptC interaction have been reported, but the precise molecular mechanisms are currently unclear [12,13]. Another peptide inhibitor, Thanatin, targeting the interaction between LptA and LptC, was also reported [14,15], and the co-crystal structure of Thanatin and LptA was solved to indicate the binding pose (PDB ID: 6GD5) [16]. However, Thanatin is an antimicrobial peptide with complex mechanisms that are not solely focused on inhibiting the interaction between LptA and LptC [14,15].

Due to the unresolved co-crystal structure of LptA and LptC, the interaction mode of these compounds remains unclear and can only be inferred using indirect evidence. Previous studies have revealed that the N-terminus of LptC extends into the inner membrane and interacts with LptB (PDB ID: 6MI7) [17]. Furthermore, it was reported that mutations in the C-terminal region of LptC, such as G153R and the Δ177–191 deletion, significantly disrupted the formation of the LptA–LptC complex [18], which indicated that the C-terminus of LptC might be the binding interface with LptA.

Given the potential application of inhibiting the interaction between LptA and LptC in antibacterial research, the current study aimed to construct and validate a model of the protein–protein interaction (PPI) between LptA and LptC based on the protein mutation data and the existing inhibitors, so that new potential inhibitors can be screened to effectively disrupt such PPI. Computational chemistry methods were primarily employed in this research to explore the binding modes and propose a specific framework for lead compound discovery.

## 2. Results and Discussion

### 2.1. The PPI Prediction of LptA and LptC

**Figure 3:**
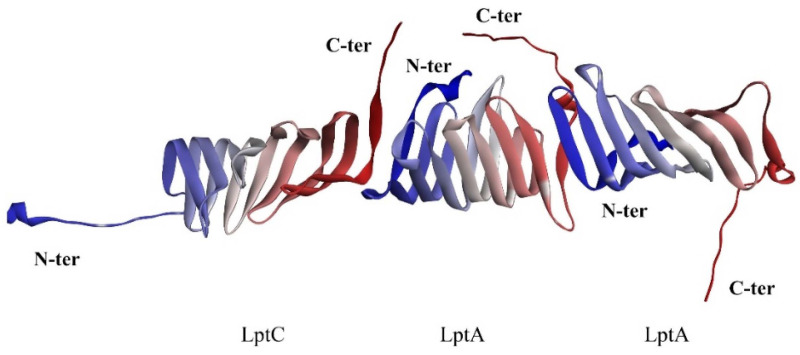
Hypothetical interaction between LptA and LptC. C-terminus: red; N-terminus: blue.

As previously described, the unique conformation of LptA and LptC, resembling a stacked arrangement, is critical for the unidirectional translocation of LPS. Integrating structural data from the crystallographic analysis of the LptA dimer (PDB ID: 2R19) and the LptA–inhibitor peptide complex (PDB ID: 6GD5), along with biochemical and biophysical insights, we propose that for the unidirectional translocation of LPS to occur, LptC must interact with LptA in a specific orientation that enables the formation of a functional channel (Figure 3). As it was reported that the N-terminal of LptC is anchored within the inner membrane, the free C-terminal of LptC can bind to the N-terminal of LptA and, subsequently, the following LptAs can sequentially connect to assemble the LPS transport channel.

**Figure 4:**
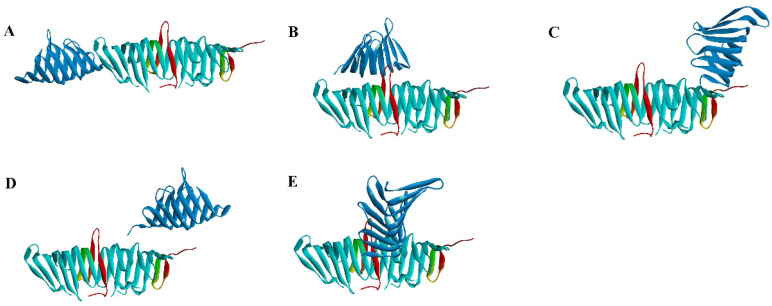
Binding conformations from the docking results. (**A**–**E**): potential binding conformation. Blue: LptA; Cyan: LptA Dimer (C-ter: Red).

In order to predict the binding pose of LptC and LptA, the Rosetta [19] protein–protein docking software was applied to dock LptC (PDB ID: 3MY2) to the LptA dimer (PDB ID: 2R19) by using a global docking approach (free dock without any binding site constraint). The overall protein energy score (the total score) and the protein interface interactions score (interface score) were calculated to evaluate the binding interactions. Among those high-scoring conformations (top 5%), both the total and interface scores exhibit a significant resemblance, and five potential binding conformations were discovered using clustering analysis (Figure 4).

**Figure 5:**
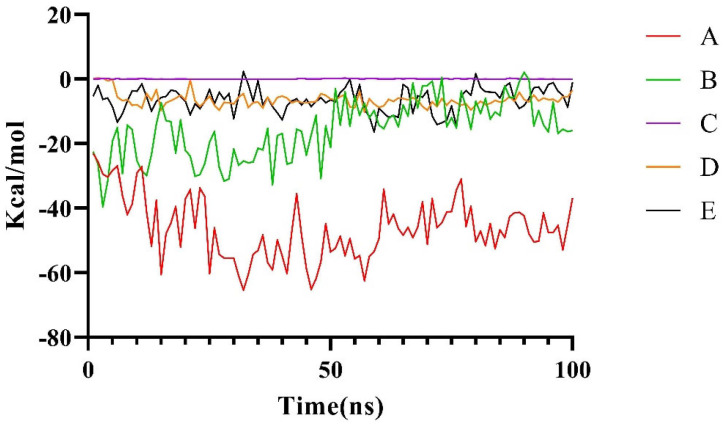
Changes in the binding free energy for local and global conformations during 100 ns.

Among these five conformations, only Conformation A is consistent with our prediction (Figure 3). To further assess the stability of the potential binding complex between the LptA dimer and LptC, a 100 ns molecular dynamics (MD) simulation was conducted for each system. The stability of these complexes was evaluated by monitoring the binding free energy fluctuations throughout the simulation (Figure 5). The results from the MD simulations and free energy calculations suggest that Binding Conformation A (Figure 4) was the most stable configuration, characterized by the significantly lower free energy compared to the alternative conformations.

**Figure 6:**
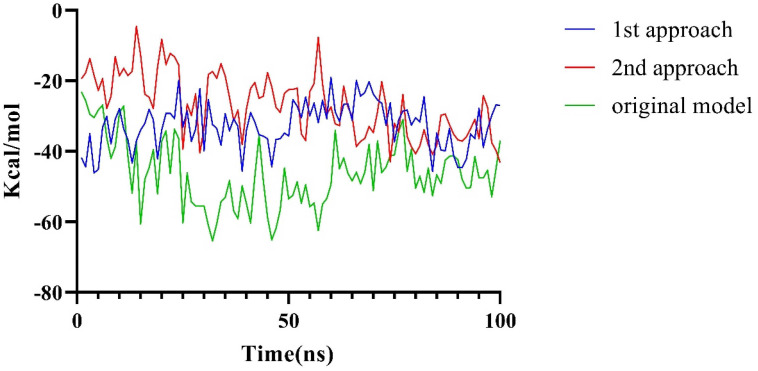
Changes in binding free energy caused by the G153R Mutation.

It was reported that mutations at the N-terminus of LptA can impede its binding with LptC [18], which indicates that the N-terminus of LptA may directly bind with LptC (consistent with Conformation A). In order to validate our model, the structural plausibility was examined by evaluating the impact of the G153R mutation of LptC based on an MD simulation. Two approaches were employed to assess the significance of the mutation. In the first approach, based on the observation that the direct mutation of G153R does not introduce protein collision, we built such a mutation and examined the binding energy loss based on a 100 ns MD simulation. In the second approach, the G153R LptC model was built, then followed by a 100 ns simulation to achieve a stable conformation. Subsequently, the complex of G153R LptC and LptA was built by using Rosetta, followed by a 100 ns MD simulation to assess changes in binding free energy. The free energy alterations (Figure 6) from both approaches exhibited a noticeable reduction in binding energy over the 100 ns simulation period. As Figure 6 shows, the significant decreases (over 10 Kcal/mol in average) in binding energy were observed in both approaches, which can validate the reliability of Conformation A.

### 2.2. Virtual Screening of PPI Inhibitors

#### 2.2.1. Molecular Docking

In order to identify novel inhibitors to disturb the PPI of LptA–LptC for potential antibacterial drug discovery, molecular docking was performed to screen Enamine’s PPI Inhibitor Library (40,640 compounds, https://enamine.net/compound-libraries/targeted-libraries/ppi-library (accessed on 20 October 2023)) and Enamine’s Diversity Library (50,240 compounds, https://enamine.net/compound-libraries/diversity-libraries (accessed on 2 April 2024)) with a total of 90,880 compounds. We extracted LptA and LptC as separate protein receptors from their respective PDB entries, 2R19 and 3MY2. The docking grid was selected according to those residues located on the protein–protein binding interface. Molecular docking calculations were performed using Vina [20].

**Figure 7:**
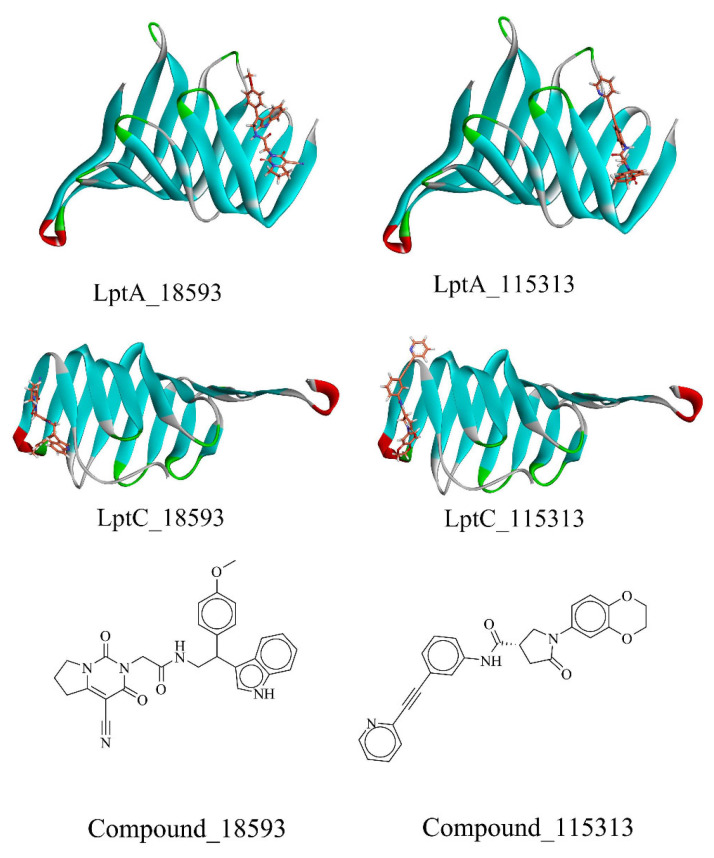
The screened compounds. Stick: compounds; Ribbon: protein.

After the dock was completed, we conducted a visual inspection of the high-scoring compounds (top 20, which are listed in the Supplementary Materials) and evaluated their binding modes at the molecular level, including hydrogen bond formation, hydrophobic interactions, and geometric coordination between molecules. The docking scores revealed that the top 10 scoring molecules all originated from the Enamine’s PPI Inhibitor Library. As shown in Figure 7, two compounds (18593 (PubChem CID: 41136112, the docking score of LptA: −8.695 kcal/mol and LptC: −9.84 kcal/mol) and 115313 (PubChem CID: 40141672, LptA: −8.774 kcal/mol, LptC: −9.85 kcal/mol)) were identified with favorable scores against both LptA and LptC, which indicated potential binding abilities to the dual targets, and were selected for further investigation.

#### 2.2.2. Molecular Dynamics Simulation

**Figure 8:**
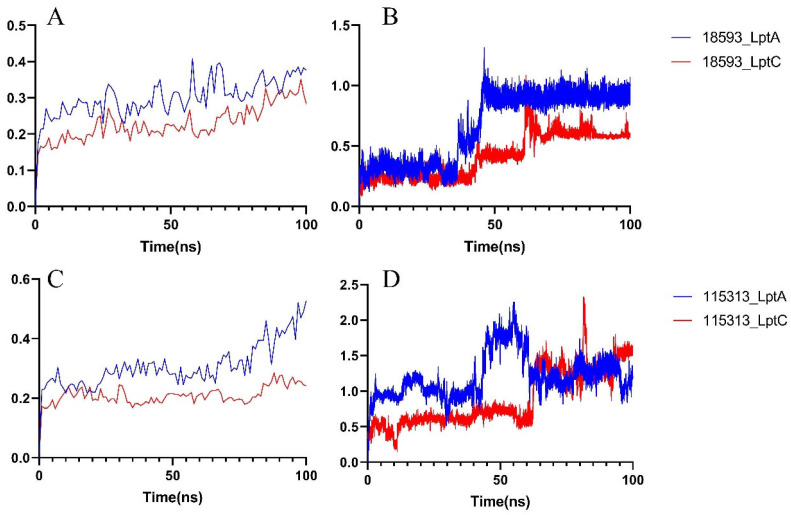
RMSD of the 18593–protein complex system. (**A**) Protein RMSD; (**B**) Ligand RMSD; RMSD of the 115313–protein complex system. (**C**) Protein RMSD; (**D**) Ligand RMSD.

In order to further study the stability of the protein–ligand interactions, a 100 ns MD simulation was performed on each system (including a selected compound with either LptA or LptC). As shown in Figure 8A, in compound 18593 with the LptA or LptC system, each protein conformation remains stable throughout the simulation period without significant conformational fluctuations. As shown in Figure 8B, the binding of compound 18593 to LptA underwent a conformational change at around 50 ns and eventually reached a stable binding pose. The binding conformation of compound 18593 with LptC experienced some changes at around 73 ns and then maintained a relatively stable conformation throughout the simulation.

As shown in Figure 8C, in comparison to compound 18593, the binding of compound 115313 causes more pronounced variations on LptA, while the LptC protein demonstrates a higher stability. As shown in Figure 8D, compound 115313 fails to stably bind to the surfaces of both proteins during the simulation.

**Figure 9:**
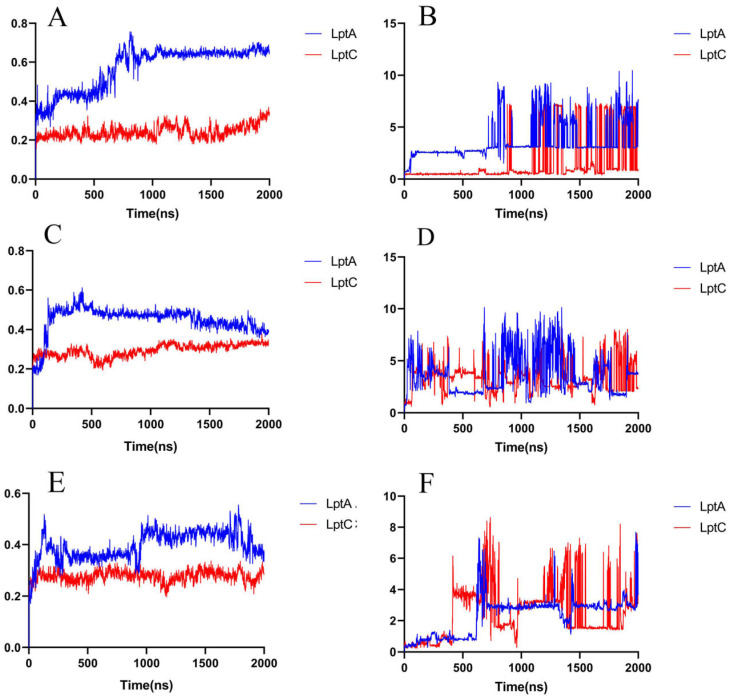
RMSD of the compounds in a 2000 ns simulation. (**A**) Protein RMSD of the 18593–protein complex system; (**B**) Ligand RMSD of the 18593–protein complex system; (**C**) Protein RMSD of the IMB-0042–protein complex system; (**D**) Ligand RMSD of the IMB-0042–protein complex system; (**E**) Protein RMSD of the IMB-881–protein complex system; (**F**) Ligand RMSD of the IMB-881–protein complex system.

The results of the MD simulations indicate that compound 18593 has the potential to be a LptA and LptC dual-targeted PPI inhibitor. In order to simulate if compound 18593 can maintain interactions with LptA or LptC for a certain period to effectively disturb the PPI, we extended the MD simulation to 2000 ns (Figure 9).

**Figure 10:**
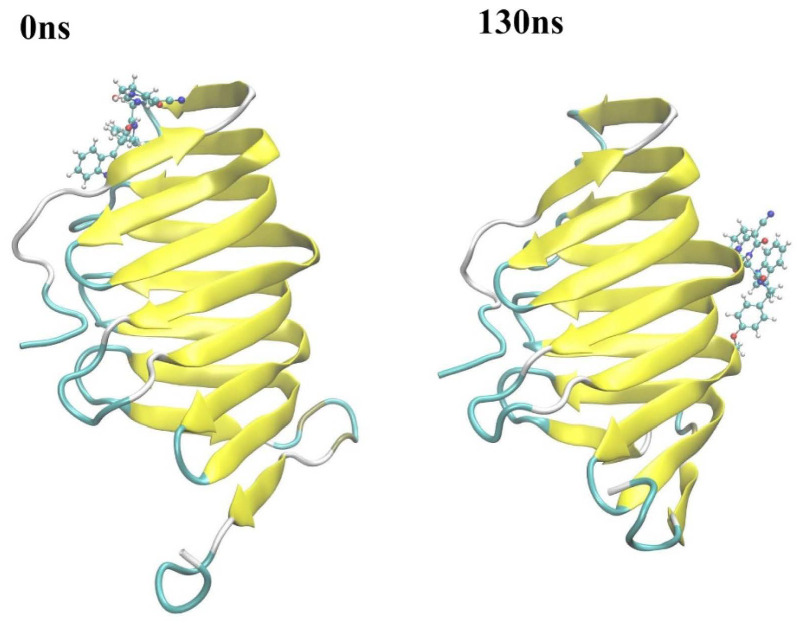
Conformational changes in compound 18593 with LptA at around 130 ns. Stick: compounds; Ribbon: protein.

At around 130 ns along the simulation trajectory of compound 18593 with LptA, the compound moved out from its original binding site (predicted by molecular docking), which comprised the residues Ile36, Arg76, Val52, His37, Ser40, and Gln43, to a new site containing the residues Gly125, Lys91, Ser110, Gly90, Gln111, and Tyr89 (Figure 10). From 810 ns to the end of the simulation, compound 18593 repeatedly dissociated and rebound to LptA at various binding sites.

In contrast, in the first 1000 ns MD simulation, compound 18593 exhibited a high stability in the predicted binding site on LptC, while LptC maintained a stable conformation as well, although compound 18593 exhibited two minor conformational changes at around 632 ns and 900 ns. From around 1200 ns to the end of the simulation, compound 18593 repeatedly dissociated and rebound to LptC (within several binding sites).

Considering the detachment and reattachment events of compound 18593 in the late stage of the simulations, the reported inhibitors IMB-881 and IMB-0042 were used as references to further validate the rationality of the screen results. Thereafter, 2000 ns MD simulations were conducted on IMB-881 and IMB-0042 with LptA or LptC complex. It can be observed that the two molecules underwent multiple cycles of dissociation and reassociation throughout the entire 2000 ns simulation (Figure 9) as well, which is consistent with their slow-binding-slow-dissociation kinetic characteristics obtained by surface plasmon resonance (SPR) [11,12,21].

The results indicate that both IMB series compounds and Compound 18953 exhibit similar binding and dissociation behaviors and may disturb the PPIs between LptA and LptC by occupying various binding sites. The results also indicated that the binding of these compounds to each target is relatively weak and unstable, but as dual target inhibitors on the same biological pathway, they may perform significant biological effects due to their synergistic effects.

#### 2.2.3. The PPI Blocking Ability of Compound 18593

**Figure 11:**
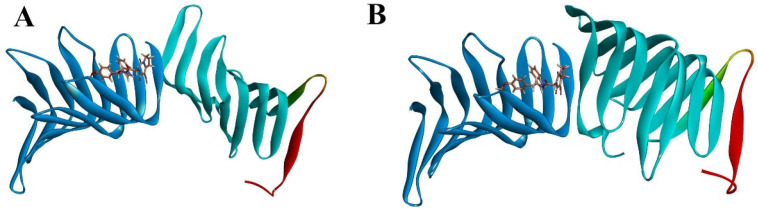
Docking results of the LptA and LptC–Compound 18593 Complex. (**A**): cluster top1; (**B**): cluster top 2. Blue: LptC; Cyan: LptA Dimer (C-ter: Red).

To further elucidate the inhibitory effect of compound 18593 on the interaction between LptA and LptC, as well as considering that the binding site of compound 18593 on LptA is distinct from the PPI interface, we conducted a docking study on the LptA to LptC–18593 complex under the same protein–protein docking conditions as described above. In the presence of compound 18593, LptA and LptC cannot form a reasonable protein–protein complex, and it is impossible to retrieve a protein–protein binding conformation that aligns with the previously predicted binding mode of the two proteins. The clustered docking conformations with the high-ranking scores are shown in Figure 11. These compellingly data support that compound 18593 may effectively disrupt the PPI between LptA and LptC.

#### 2.2.4. ADMET Predictions

We utilized computational models (ADMETlab 2.0) to predict the absorption, distribution, metabolism, excretion, and toxicity (ADMET) characteristics of compound 18593, which are crucial for assessing its drug-like potential. Compound 18593 satisfies the Lipinski’s Rule of Five: molecular weight (MW) ≤ 500 Da, logP < 5, number of hydrogen bond donors (nHBD) ≤ 5, number of hydrogen bond acceptors (nHBA) ≤ 10, and total polar surface area (TPSA) < 140 Å^2^.

Additionally, compound 18593 successfully passed both the Pfizer Rule, which assesses oral bioavailability, and the PAINS (Pan-Assay Interference Compounds) tests, which screen for chemical substructures that are likely to interfere with biological assays, further indicating its potential drug-like properties.

Furthermore, predictions indicate that compound 18593 has an MDCK (Madin-Darby Canine Kidney) permeability of 0.000011 cm/s and exhibits a bioavailability greater than 90% when administered at a 30% dose. This suggests that compound 18593 undergoes effective absorption in the human intestinal tract.

In the rat oral acute toxicity prediction, compound 18593 exhibits lower oral toxicity. However, it tests positive for hepatotoxicity in two liver injury prediction models (Hepatotoxicity (H-HT) and Drug-Induced Liver Injury (DILI)), and yields positive results in two carcinogenicity prediction assays (AMES Toxicity and a general carcinogenicity test). This toxicity is acceptable for a leading compound and can usually be effectively improved by following the chemical structure optimization process.

**Figure 12:**
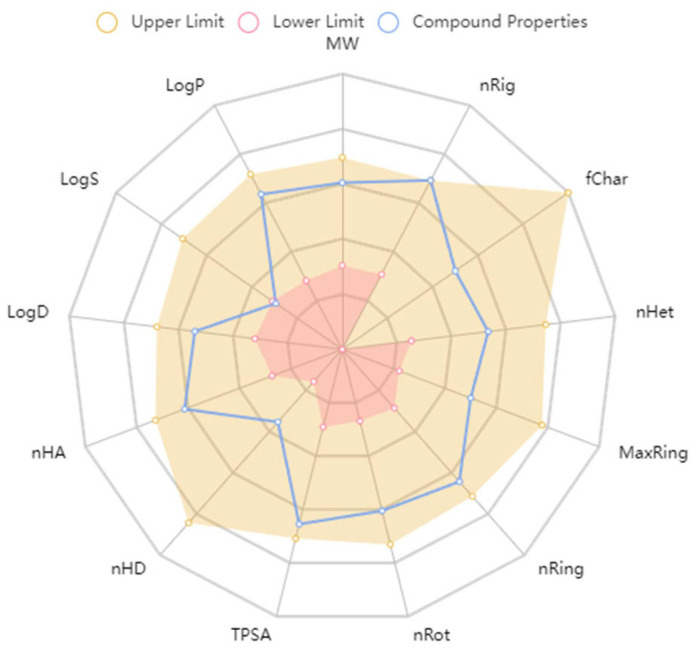
The ADMETlab 2.0 drug space.

ADMET predictions affirm that compound 18593’s physicochemical properties align with the drug-like space as defined by the ADMETlab 2.0 platform, which provides a comprehensive evaluation of ADMET properties (refer to Figure 12).

## 3. Materials and Methods

The experimental procedures were performed with two Intel Xeon Gold 5320 CPU @ 2.00 GHz processor (Intel Corporation, Santa Clara, CA, USA), using the Linux tc4600v4 operating system and four NVIDIA A100 graphics card (NVIDIA Corporation, Santa Clara, CA, USA).

### 3.1. Protein Preparation

The proteins investigated in this study were uniformly derived from *Escherichia coli*, the organism under research. The X-ray crystallographic structures of LptA (PDB ID: 2R19, resolution: 2.16 Å, r-value free: 0.271) and LptC (PDB ID: 3MY2, resolution: 2.20 Å, r-value free: 0.222) were obtained from the Protein Data Bank [22], and the resolutions of each amino acid were ensured to reach sufficient accuracies. The water molecules, the co-crystallized ligands, and unnecessary ligand groups were removed from the protein structure. The RosettaCM was then employed to complete missing segments within the protein structures. Both the downloaded single-crystal structures and the predicted structures from Alphafold2 served as templates for modeling [23,24]. The reconstructed LptC protein was further optimized using the OPLS4 force field to ensure stability in the OM region [23,25]. Finally, the PDB structures were converted to the PDBPT format for subsequent docking. Site-directed mutations of the amino acids mentioned in the study were executed using Rosetta’s Mutfile [26].

### 3.2. Ligand Preparation

The Protein–Protein Interaction Library and the Diversity Library of Enamine Ltd was employed for molecular screening. The Protein–Protein Interaction Library consists of 40,640 diverse compounds specifically designed to target protein–protein interactions. Furthermore, the library’s design incorporates several specific recognition patterns, such as α-helices, β-sheets, PDZ domains, PBD domains, and bromodomains, which mimic the structural domains found within proteins themselves. The Diversity Library consists of 50,240 compounds, which are aimed at bringing the latest compound skeleton. The compounds in the Diversity Library are meticulously selected to represent the molecular diversity of the HTS, Advanced Library, and Premium Libraries of Enamine Ltd.

All molecules were split by using the OpenBabel program (version 3.0.0) [27,28]. All possible stereoisomers for each molecular structure were computed. After the generation of isomers, the molecules and their isomers were sorted and renumbered. Subsequently, they were hydrogenated and energy minimized using a high-precision MMFF94 force field [28]. The 3D structures ultimately generated were used in the molecular docking studies.

### 3.3. Molecular Docking

Autodock Vina 1.2.3 was employed for all molecular docking work in this study. The exhaustiveness was set to 64, and the maximum energy difference from the best pose was set to 3 [20]. Different docking parameters were configured for various docking scenarios.

For IMB-881 and IMB-0042, as no crystallographic data were available for the interaction between the LptA and LptC proteins, in order to maximize the coverage of the inhibitor binding possibilities, the output num_mode was set to 120. Additionally, the docking grid was set to cover a large cubic space on the protein surface. For LptA, a grid box centered at x = 70.856, y = 74.191, and center_z = 77.016, with dimensions of 66 × 50 × 50 Å, effectively covered the protein surface. For LptC, a grid box centered at x = 112.865, y = 78.556, and center_z = 72.04, with dimensions of 50 × 50 × 50 Å, effectively covered the protein surface.

In the virtual screening workflow, both the LptA and LptC isolated from the previously established PPI model were used as the protein receptors, and the interfaces between them were chosen as the binding sites for molecular docking. Once the molecular docking was finished, LptC/LptA was aligned back into each system to observe whether the docked molecule could directly influence the PPI. To ensure a comprehensive exploration of conformations, the docking grid was designed to extend beyond the binding interface. Different from the IMB series compounds docking settings, only 9 conformations were filtered during the virtual screening process. For LptA, a grid box centered at x = 73.185, y = 65.144, and center_z = 64.552, with dimensions of 30 × 30 × 30 Å, effectively encompassed the binding interface with LptC. For LptC, a grid box centered at x = 82.976, y = 59.335, and center_z = 71.692, with dimensions of 24 × 24 × 24 Å, thoroughly covered the binding interface with LptA.

Protein–protein docking was conducted using the Rosetta software, version 2022.12 [29]. Both the local dock and the global dock generated 50,000 structures each, and the default settings were used. In the validation of compound 18593’s disruption of PPI, both proteins were pulled apart by a certain distance to ensure that the program would not encounter errors due to atoms being too close. Simultaneously, the molecule was fixed to LptC, as a part of LptC during the docking process.

### 3.4. Molecular Dynamics Simulations

MD simulations were performed using the docking results to enable the further analysis of the binding of protein–ligand complexes. All MD simulations were performed using the GROMACS package, version 2023.2 [30], and the AMBER99SB-ILDN force field was employed for the protein [31], while the general AMBER force field (GAFF) was generated by Sobtop for the binding molecule [32] and TIP3P as the water solvation model. A cube (with the distance between the protein and the edge of box greater than 0.8 nm) was used as the MD box [33,34]. A water model was added to the container at a density of 1000 g/L, and an adequate number of water molecules was replaced by counter ions (Na^+^ and Cl^−^) to achieve an electrically neutral system. The system was first minimized through the steepest descent minimization approach [35], followed by the restricted MD simulations to release any restraints. In this restricted MD, the temperature of the system was slowly increased to 298.15 °C by a 500 ps. Lastly, the free dynamic simulations were performed using the Verlet algorithm [35]. The integration step was set at 0.002 ps. The simulations were performed in an isothermal isobaric regime at 298.15 K and under 1 bar pressure, with temperature and pressure controlled with the V-rescale and Parrinello–Rahman methods [36], respectively, and PBC (Periodic Boundary Condition) was enabled. The simulation times were extended based on the mission needs. The RMSD (Root Mean Squared Deviation) was calculated for protein–protein and protein–molecules interactions. MD trajectories were viewed using VMD software, version 1.9.4 [37].

The binding free energy of the protein–ligand complex was performed by gmx_MMPBSA package using the molecular mechanics/Poisson–Boltzmann (generalized Born) surface area method [38,39].

### 3.5. ADMET Predictions

We used ADMETlab 2.0 to perform the ADMET prediction, which is a model based on 30 different datasets consisting of 280,000 molecules, and the R^2^ (the coefficient of determination) of each model was greater than 0.95, ensuring that the prediction result was statistically significant [13]. The ADMET prediction software (ADMETlab 2.0) is capable of predicting an array of properties, including 17 physicochemical characteristics, 13 medicinal chemistry attributes, 23 ADME (Absorption, Distribution, Metabolism, and Excretion) properties, 27 toxicity endpoints, and applying 8 toxicophoric rules encompassing 751 substructures. During the computational phase, it effectively screens out compounds with inferior ADMET properties.

## 4. Conclusions

It is believed that targeting the interactions between LptA and LptC could be a potential strategy to discover new anti-G- bacterial drugs. However, due to a lack of co-crystal structures, the detailed LptA–LptC interactions are unknown. In the current manuscript, we employed the computer-aided drug design (CADD) methodology to build a model to investigate the interactions between LptA and LptC. Such a computational model was validated by reproducing the protein mutagenesis according to the experimental data.

The potential inhibitors of the LptA–LptC protein–protein interactions were identified through molecular docking and MD simulations, among which is the compound 18593, which demonstrates effective binding abilities to both LptA and LptC, thereby showing a potential ability to disrupt the interactions between these two proteins. The binding poses and binding affinities of the screened molecules were then evaluated and compared with those of the previously reported inhibitors. Concurrently, the binding and dissociation processes of the compounds were comprehensively simulated and sampled. It was indicated that both the IMB series compounds (known inhibitors) and compound 18593 exhibited similar binding and dissociation behaviors, and compound 18593 exhibited a better binding performance compared to the IMB series compounds. The ADMET predictions indicated that the identified compound possesses favorable drug-like properties, and thus compound 18593 emerged as a promising lead candidate for further investigation.

## Data Availability

The data presented in this study are available on request from the corresponding author.

## Supplementary Materials

The following supporting information can be downloaded at: https://www.mdpi.com/article/10.3390/molecules29081827/s1, Top 20 high-scoring compounds.
